# Synthesis of Cu-doped V_2_O_5_ thin films with improved optical and CO_2_ gas sensing

**DOI:** 10.1039/d5ra07026k

**Published:** 2026-01-02

**Authors:** Khaled Abdelkarem, Rana Saad, Mohamed Shaban, Adel M. El Sayed

**Affiliations:** a Department of Physics, Chonnam National University Gwangju 61186 Republic of Korea oldfighter.khaled123@gmail.com; b Department of Physics, Faculty of Science, Beni-Suef University Beni Suef 62511 Egypt ranasaad811@gmail.com; c Department of Physics, Faculty of Science, Islamic University of Madinah P. O. Box: 170 Madinah 42351 Saudi Arabia mssfadel@aucegypt.edu; d Department of Physics, Faculty of Science, Fayoum University El-Fayoum 63514 Egypt ams06@fayoum.edu.eg

## Abstract

This study provides a comprehensive investigation of Cu-doped vanadium oxide (V_2_O_5_) thin films prepared *via* a sol–gel/spin-coating method, correlating dopant-induced structural and optical modifications with improved CO_2_ sensing performance at room temperature. XRD confirmed the incorporation of Cu into the V_2_O_5_ lattice without secondary phase formation, while FE-SEM revealed a morphological transition from nanoplates to nanobelts upon Cu-doping. EDX verified uniform elemental distribution, and UV-Vis measurements indicated a reduced optical band gap, consistent with enhanced charge transport. FTIR spectra exhibited characteristic V–O vibrations, along with CO_2_-related absorption bands, indicating favorable surface interactions. Gas sensing experiments demonstrated that Cu incorporation significantly improved sensitivity, response/recovery times, and selectivity. At 8880 ppm CO_2_, the 10 at% Cu-doped V_2_O_5_ films achieved a response of 40.7% with fast response (3.83 min) and recovery (3.3 min) times, excellent repeatability, and stable operation over 30 days. These findings establish 10 at% Cu-doped V_2_O_5_ thin films as a promising, low-cost material for efficient room-temperature CO_2_ detection.

## Abstract 2

This study examines sol–gel/spin-coated Cu-doped vanadium oxide (V_2_O_5_) thin films and their structural and optical changes, which improve CO_2_ sensing at ambient temperature.

## Graphical abstract

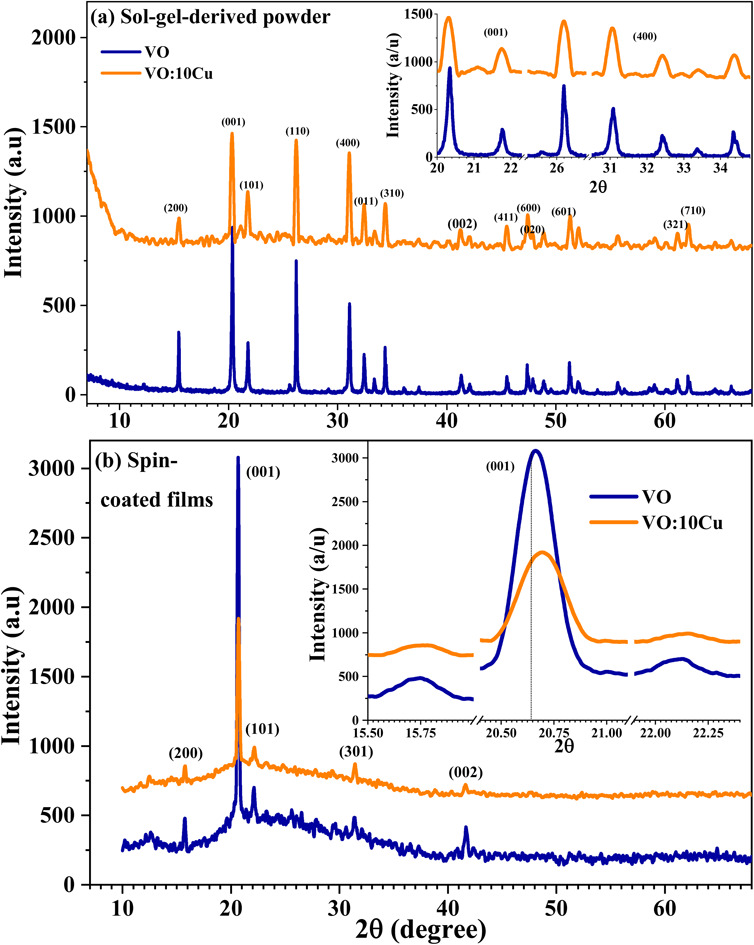

## 1 Introduction

Monitoring carbon dioxide (CO_2_) concentrations is vital in areas such as indoor air quality control, industrial safety, and environmental protection. Elevated CO_2_ levels in confined spaces can impair human health, while the steady rise of atmospheric CO_2_ continues to drive global climate change. These concerns highlight the need for gas sensors that combine high sensitivity and selectivity with low cost and reliable operation under ambient conditions.^1,2^ Metal oxide semiconductors (MOS) are among the most widely studied materials for gas detection due to their chemical stability, tunable electrical properties, and strong surface reactivity.^3–6^ Within this class, vanadium pentoxide (V_2_O_5_) has drawn particular attention. Its layered orthorhombic structure, variable oxidation states (V^5+^/V^4+^), and high chemisorption capability make it highly responsive to adsorbed gas species.^7,8^ Moreover, V_2_O_5_ possesses notable catalytic activity and a relatively narrow band gap (∼2.3 eV), allowing efficient gas detection at low operating temperatures—an essential feature for energy-efficient sensors. Despite these advantages, pristine V_2_O_5_ exhibits only moderate CO_2_ sensing behavior. Recent research has shown that doping with transition metals can significantly improve their performance by tailoring the electronic structure, increasing oxygen vacancy density, and facilitating charge carrier transport.^9–11^ Among various dopants, copper (Cu) is especially promising due to its ability to stabilize surface states, enhance electrical conductivity, and promote active sites for gas adsorption.^12^ Although several studies have reported CO_2_ sensing using V_2_O_5_-based materials, most of these works have focused on bulk powders, glass–ceramic composites, or quartz crystal microbalance (QCM)-type devices. In contrast, this study offers a comprehensive investigation of Cu-doped V_2_O_5_ thin films prepared *via* a low-temperature sol–gel/spin-coating route, emphasizing the correlation between Cu-induced structural, optical, and electronic modifications and their influence on room-temperature CO_2_ sensing behavior. The obtained thin films exhibit phase-pure α-V_2_O_5_, controlled nanostructure, and reduced crystallite size. The spin-coated Cu-doped V_2_O_5_ thin films that operate at room temperature, quantifying a ∼7× sensitivity enhancement with faster response/recovery and month-long stability, establishing a link between structure and properties among (001) preferred orientation, *E*_g_ narrowing, and oxygen-vacancy-mediated adsorption/charge transfer that underpins selectivity, and narrowed optical band gap, which collectively lead to enhanced sensitivity. Therefore, this work highlights Cu-doped V_2_O_5_ thin films as promising, low-cost, and energy-efficient materials for ambient CO_2_ detection, providing new insights into dopant-driven performance enhancement in vanadium oxide systems.

## 2 Materials, film preparation, and experimental work

### 2.1 Chemicals

V_2_O_5_ powder (molecular weight ≈ 181.9 g mol^−1^, purity ≥99.6%, Merck) was used as the precursor for vanadium oxytrichloride (VOCl_3_) synthesis. Concentrated hydrochloric acid (HCl, 36%, ∼36.5 g mol^−1^, Merck) served as the solvent for V_2_O_5_ dissolution. Oxalic acid ∼126.1 g mol^−1^ acted as a stabilizing (chelating) agent, while Cu-acetate monohydrate (Cu(CH_3_COO)_2_·H_2_O, ≈199.7 g mol^−1^, purity ≈98%, supplied by Panreac) was employed as the dopant precursor for the 10 at% Cu-doped V_2_O_5_ films.

### 2.2 Synthesis procedure

Bulk V_2_O_5_ powder was dissolved in concentrated HCl under stirring at 100 °C for 2 h, producing a green VOCl_3_ precipitate. A 2.0 g portion of this precipitate was redissolved in 50 mL of ultrapure water, ∼1.6 g of the stabilizing agent was added, and further stirring at 60 °C for 1 h. The resulting gel was heated at 100 °C for 3 h to remove the excess water and subsequently calcined in air at 445–455 °C for 2 h to yield nanostructured V_2_O_5_ powder. For the Cu-doped material, the same procedure was followed with the addition of ∼0.6 g of copper acetate monohydrate to the oxalic acid solution before gel formation. To fabricate thin films, the as-prepared pure and 10 at% Cu-doped V_2_O_5_ powders were dispersed in a dilute chitosan solution; it was used solely as a binder to improve film adhesion and mechanical stability during spin-coating, ensuring uniform deposition of the V_2_O_5_-based nanoparticles on the glass substrate. Its concentration was kept very low and identical for all samples (pure and Cu-doped), minimizing any chemical interaction or electronic contribution to the sensing behavior. Although chitosan is an insulating biopolymer, its presence is not expected to significantly affect the film's electrical conductivity because the continuous V_2_O_5_ network dominates charge transport. Moreover, any influence on gas adsorption would be indirect, limited to providing minor surface hydrophilicity that stabilizes the film morphology without altering the sensing mechanism; then deposited onto pre-cleaned glass substrates *via* casting or spin-coating. The films were dried at 120 °C for 2 h to ensure adhesion and structural stability.

### 2.3 Characterization and room-temperature CO_2_ sensing analysis

The structure, morphology, composition, and optical properties of the pure and 10 at% Cu-doped V_2_O_5_ thin films were investigated using complementary techniques. X-ray diffraction (XRD) patterns were recorded with a Smart Lab diffractometer (RIGAKU) using Cu Kα radiation (*λ* = 0.1544 nm) over a 2*θ* range of 10–80° with a step size of 0.02°, enabling phase identification and assessment of Cu incorporation into the V_2_O_5_ lattice. The morphology of the powder obtained by the sol–gel route was examined by field-emission scanning electron microscopy (FE-SEM, QUANTA 200F), and elemental (chemical) composition was verified using energy-dispersive X-ray spectroscopy (EDX) attached to the same system. These analyses confirmed the nanostructured features of the powder and films and the successful incorporation of Cu dopants. Bonding and vibrational characteristics were studied using attenuated-total reflectance Fourier-transform infrared spectroscopy (ATR-FTIR, Vertex70, Bruker). Spectra were collected in the 4000–400 cm^−1^ range, with glass substrate backgrounds recorded separately to isolate the film response. Optical properties were measured using UV-Visible spectroscopy (JASCO V-630) across 200–1600 nm with a resolution of 2 nm and accuracy of ±0.2 nm. These measurements provided information on transmittance, absorbance, absorption coefficient, reflectance, refractive index, and optical band gap variations upon Cu doping. The CO_2_ sensing performance was evaluated using a custom-built setup based on a standard MOS sensor configuration. A sealed 1.0 L glass chamber was employed, fitted with electrical feedthroughs and a side inlet for gas injection. High-purity CO_2_ (≈99.9999) and dry air were supplied from calibrated cylinders, and flow rates were precisely controlled by digital mass flow controllers (Alicat MC-500SCCM-D, Smart Track, Sierra Instruments). Thin films were contacted with silver paste electrodes to ensure stable ohmic electrical connections. Electrical response, including *I*–*V* characteristics and dynamic sensing behavior under varying CO_2_ concentrations, was measured with a Keithley 2450 source-measure unit (Tektronix). All gas sensing experiments were performed at room temperature to demonstrate practical ambient operation.

## 3 Result and discussion

### 3.1 XRD, SEM, EDX, cross-sectional SEM measurements, and FTIR analyses

**Fig. 1:**
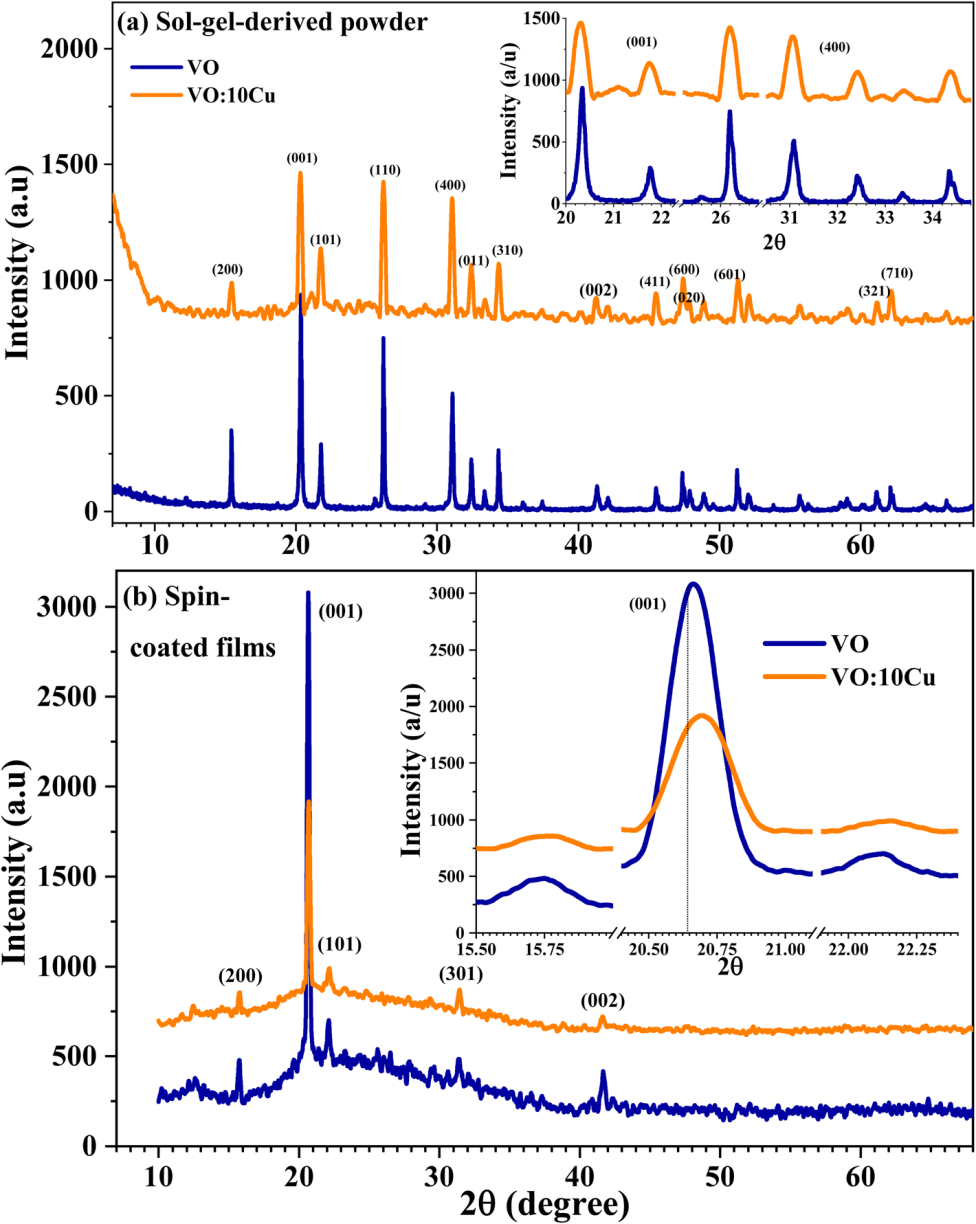
XRD spectra (patterns) of the pure and 10 at% Cu-doped V_2_O_5_: (a) powder and (b) films. The inset shows the broadening and shift for the most intense peak.

The crystalline structures of the sol–gel-derived pure and 10 at% Cu-doped V_2_O_5_ thin films were examined using X-ray diffraction (XRD), and the patterns are shown in Fig. 1a and b. Multiple sharp peaks were detected within the scanned 2*θ* range, confirming the polycrystalline nature of the materials. These reflections correspond to the orthorhombic α-phase of V_2_O_5_ in agreement with JCPDS card no. 89-0612.^7,8^ The phase is indexed to space group *Pmmn* (No. 59), with lattice parameters *a* = 11.4980 Å, *b* = 3.5450 Å, *c* = 4.3450 Å, and a unit cell volume of 177.104 Å^3^. No secondary peaks from vanadium suboxides or copper oxides were observed, indicating phase purity and successful Cu incorporation into the V_2_O_5_ lattice. Compared with solid-state or combustion routes that often require high annealing temperatures (≥600 °C) and may yield secondary phases such as CuV_2_O_6_ or β-Cu_0.55_V_2_O_5_,^13,14^ the present sol–gel approach and spin coating produced phase-pure α-V_2_O_5_ powder and films at a lower annealing temperature of 450 °C. The use of bulk V_2_O_5_ as a precursor and oxalic acid as a stabilizing agent facilitated pure phase formation while minimizing energy consumption. Cu incorporation influenced peak intensity and caused subtle shifts in position without altering the overall α-phase structure.

For both pure and Cu-doped V_2_O_5_ powder and films, the (001) plane exhibited a preferred orientation, consistent with the formation of layered orthorhombic α-V_2_O_5_.^15^ This preferred growth and crystallite ordering along the (001) direction is enhanced upon turning the powder into films, where the intensity of the (001) plane strongly improved at the expense of other directions, as seen in Fig. 1b. This behavior is consistent with previous findings that the preferred growth direction in Cu-doped V_2_O_5_ nanorods shifted from (110) to (001).^16^ The decrease in the (001) peak intensity together with the increase in the FWHM (β) for the undoped V_2_O_5_ film arises from its lower crystallinity and larger structural disorder compared with the Cu-doped film. During spin-coating and annealing, undoped V_2_O_5_ tends to form micro-aggregated grains with partial misorientation, leading to weaker preferred orientation and broader diffraction peaks. Upon Cu incorporation, Cu^2+^ ions substitute for V^5+^ in the lattice, introducing slight lattice strain and additional oxygen vacancies that act as nucleation centers. These centers promote more uniform crystal growth and improved ordering along the direction (001). As a result, the Cu-doped film exhibits higher peak intensity and narrower β, indicating enhanced crystallinity.

The crystallite size *C*_s_ was estimated using the Scherrer equation:1
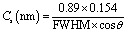
where 0.89 is the Scherrer constant, *λ* = 0.154 nm is the CuKα wavelength, and *β* is the full width at half maximum (FWHM) of the peak. Using this method, the average *C*_s_ of the powder samples decreased from 55.2 ± 1.6 nm for the pure V_2_O_5_ to 49.2 ± 3.5 nm for 10 at% Cu-doped sample, while the average *C*_s_ of the films decreased from 65.2 ± 2.6 nm for V_2_O_5_ to 59.2 ± 5.1 nm for the Cu-doped film. This reduction indicates a modest decrease in crystallinity, supported by lower peak intensities, and may also be influenced by the use of chitosan during film deposition, which can suppress grain growth. Peak position analysis revealed contrasting behavior between powders and films. While Cu-doped powders typically exhibit leftward shifts in the (001) and (101) peaks (see the inset of Fig. 1a) due to substitution of V^5+^ ions (*r* = 0.054 nm) by larger Cu^2+^ ions (*r* = 0.071 nm), which increases interlayer spacing,^17^ the Cu-doped films in this study displayed a rightward shift of the (001) peak compared with undoped films (the inset of Fig. 1b). This unusual effect is attributed to strain, local bonding changes, or film–substrate interactions during crystallization.^18^ Both pure and Cu-doped films remained polycrystalline, dominated by the strong reflection at 2*θ* = 20.68°, consistent with (001) orientation. The refined lattice parameters for the undoped film were *a* = 11.4992 Å, *b* = 4.3701 Å, *c* = 3.5625 Å, with a unit cell volume of 179.03 Å^3^, in agreement with the α-V_2_O_5_ orthorhombic structure.

The absence of distinct Cu or CuO diffraction peaks in the XRD pattern of the 10 at% Cu-doped V_2_O_5_ film indicates that Cu ions are successfully incorporated into the V_2_O_5_ lattice rather than forming separate crystalline phases. The amount of Cu is relatively small and below the detection limit required to produce independent diffraction peaks. Instead, the Cu atoms occupy substitutional or interstitial sites within the V_2_O_5_ lattice, which results only in minor peak shifts and broadening rather than new reflections. This lattice incorporation is further supported by EDX analysis, which confirmed the presence of Cu in the material, and by the absence of any secondary phase peaks (such as CuO or Cu_2_O) in the XRD pattern. Such behavior is commonly reported for transition-metal-doped V_2_O_5_ systems where the dopant concentration is moderate and homogeneously distributed. Overall, Cu doping in thin films reduced crystallite size, altered preferential orientation, and induced peak shifts linked to strain effects. These structural modifications are expected to enhance surface reactivity and oxygen vacancy density, providing favorable conditions for the improved CO_2_ sensing performance discussed in later sections.

**Fig. 2:**
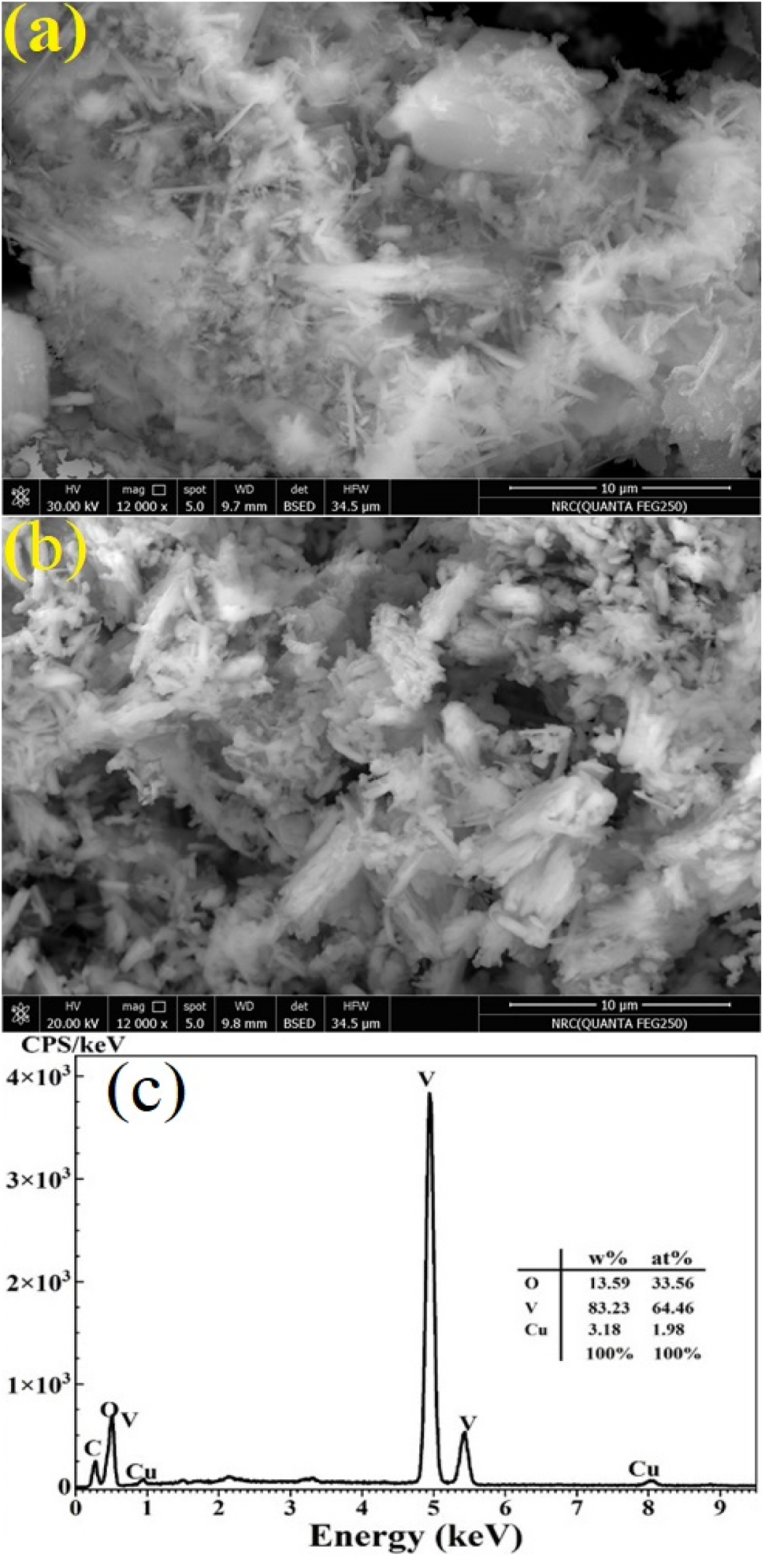
(a and b) FE-SEM images for pure, and 10 at% Cu-doped V_2_O_5_ nanostructures, (c) EDX spectrum of 10 at% Cu-doped V_2_O_5_.

The morphology of the sol–sol–gel-derived powders was examined by FE-SEM, and the results are shown in Fig. 2a and b. The pure V_2_O_5_ displayed a relatively compact surface, while the 10 at% Cu-doped V_2_O_5_ film exhibited a textured microstructure composed of densely packed nanorods and micro-baton-like features. These structures were randomly oriented within layered domains, providing a high surface-to-volume ratio that is favorable for gas adsorption and diffusion during sensing. The improved connectivity between grains also suggests efficient electron transport pathways, an important factor for enhancing sensor response. The increase in particle size and formation of voids in the 10 at% Cu-doped V_2_O_5_ film arises from Cu-induced lattice strain and structural rearrangement during the sol–gel and annealing processes. When Cu^2+^ ions substitute for V^5+^ in the V_2_O_5_ lattice, the difference in ionic radii (Cu^2+^ = 0.071 nm; V^5+^ = 0.054 nm) causes local lattice distortion and nonuniform grain coalescence. This promotes partial grain growth and the appearance of intergranular voids as the material relaxes to minimize strain energy. Additionally, during thermal treatment, Cu incorporation enhances diffusion and localized densification, leading to uneven shrinkage between adjacent grains. This results in the formation of small voids or pores among agglomerated particles, which increase the surface area and facilitate gas adsorption.

The chemical composition and homogeneity of the powders were investigated using the EDX technique. Fig. 2c presents the EDX spectrum of the Cu-doped V_2_O_5_ nanostructure, confirming the presence of the main elements V, O, and Cu. Characteristic signals were detected at 0.525 keV (O K_α1_), 0.51 keV (V L_α1_), 4.95 keV (V K_α1_), 5.43 keV (V K_β1_), and 0.93, 8.0, and 8.9 keV (Cu L_α1_, K_α1_, and K_β1_, respectively). A minor peak at ∼0.28 keV originates from the carbon support grid. No additional peaks corresponding to impurities were observed, confirming the chemical purity of the films. The measured atomic ratio [O]/[V] for the Cu-doped V_2_O_5_ nanostructure was ∼33.58/64.46, slightly lower than the ideal stoichiometry, suggesting the presence of oxygen vacancies introduced by Cu incorporation. Such oxygen deficiency is consistent with structural findings and plays a key role in promoting surface reactivity and enhancing CO_2_ sensing performance. ThisFr oxygen deficiency created by Cu incorporation plays a crucial role in enhancing CO_2_ sensing performance. When Cu^2+^ substitutes for V^5+^ in the V_2_O_5_ lattice, charge compensation occurs through the formation of oxygen vacancies. These vacancies act as active adsorption and reaction sites for gas molecules, increasing the surface reactivity of the film. During sensing, the oxygen vacancies facilitate the adsorption of oxygen species (O_2_^−^, O^−^) on the surface, which readily interact with incoming CO_2_ molecules. This interaction modulates the charge carrier concentration by trapping or releasing electrons, resulting in a larger change in resistance and thus higher sensor response. Therefore, the presence of oxygen vacancies directly enhances electron exchange, adsorption kinetics, and overall CO_2_ sensitivity.

Cross-sectional SEM measurements revealed that the pure V_2_O_5_ film had an average thickness of 8.6 ± 0.8 µm, whereas the 10 at% Cu-doped V_2_O_5_ film exhibited a reduced thickness of 5.36 ± 0.55 µm, as seen in Fig. S1. This reduction is attributed to the influence of Cu^2+^ ions on the sol–gel chemistry, where Cu modifies the viscosity and polymeric network of the precursor solution, leading to a thinner deposited layer during spin coating, while also promoting greater densification and shrinkage during thermal treatment due to enhanced cross-linking and the formation of oxygen-vacancy-driven lattice relaxation. Film thickness plays a critical role in chemiresistive gas sensing, as thicker films generally display longer gas-diffusion paths, higher bulk resistance, and lower effective surface-to-volume ratios, which can suppress sensitivity and slow down response/recovery behavior. Thinner films, by contrast, allow faster gas adsorption and desorption and provide more accessible active sites, although extremely thin films may exhibit poor mechanical stability or incomplete electrical continuity. The thickness values obtained in this study, therefore, represent a practical balance between structural integrity, continuous conduction pathways, and adequate surface area to ensure reliable and responsive CO_2_ sensing performance.

**Fig. 3:**
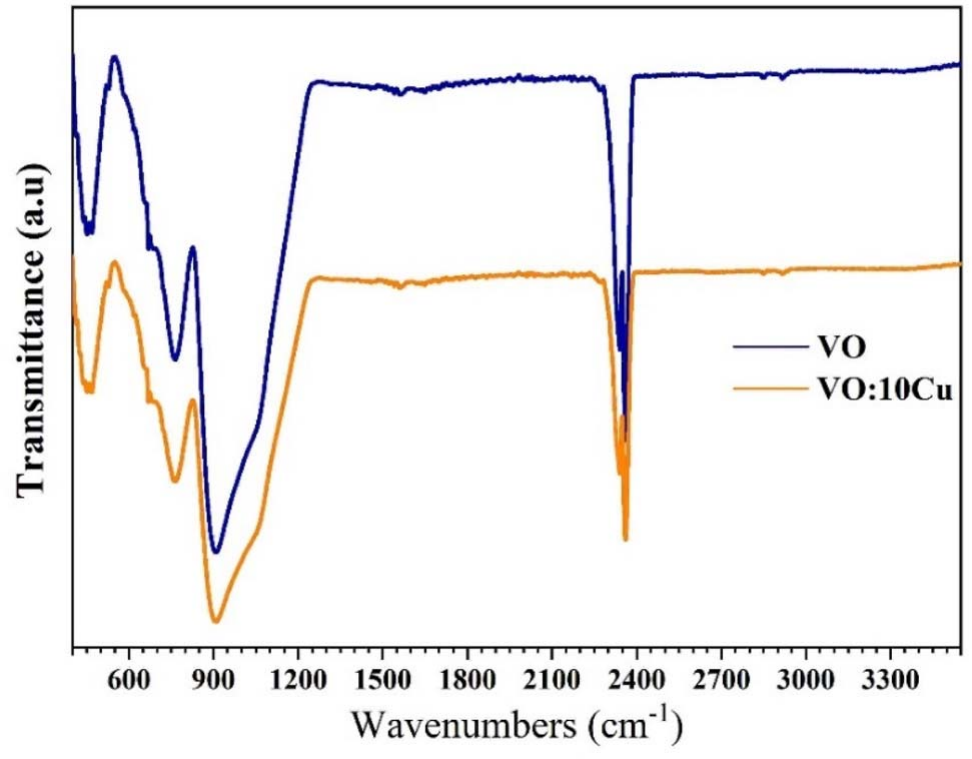
ATR-FTIR spectra of the pure and 10 at% Cu-doped V_2_O_5_ films obtained by casting and spin-deposition.

The vibrational properties of the pure and 10 at% Cu-doped V_2_O_5_ films were examined using ATR-FTIR (Fig. 3). Both samples exhibit characteristic bands below 1000 cm^−1^, corresponding to metal–oxygen vibrations. A strong absorption near 900 cm^−1^ is assigned to asymmetric V–O–V stretching, while a weak shoulder around 1050 cm^−1^ corresponds to terminal V=O stretching. The V–O–V (symmetric) mode appears at ∼760 cm^−1^, and the ∼450 cm^−1^ band is attributed to V–O bending vibrations. These assignments confirm the layered orthorhombic α-V_2_O_5_ phase, in agreement with the XRD results. A broad feature at ∼2350 cm^−1^ was observed in both spectra, attributed to adsorbed CO_2_ from the ambient atmosphere,^19^ confirming the surface activity of the films toward CO_2_ interaction. Compared with the undoped film, the Cu-doped film showed reduced intensity in the main vibrational bands, indicating a decrease in crystallinity and local distortion of V–O bonds. These effects are consistent with a smaller crystallite size and increased disorder detected in XRD. A broad band around 3350 cm^−1^, attributed to O–H stretching, appeared after irradiation, indicating enhanced water adsorption capacity. Together, these features highlight that Cu doping modifies the vibrational environment and enhances surface reactivity, both of which support improved gas interaction.

### 3.2 UV-Vis analyses for the films

**Fig. 4:**
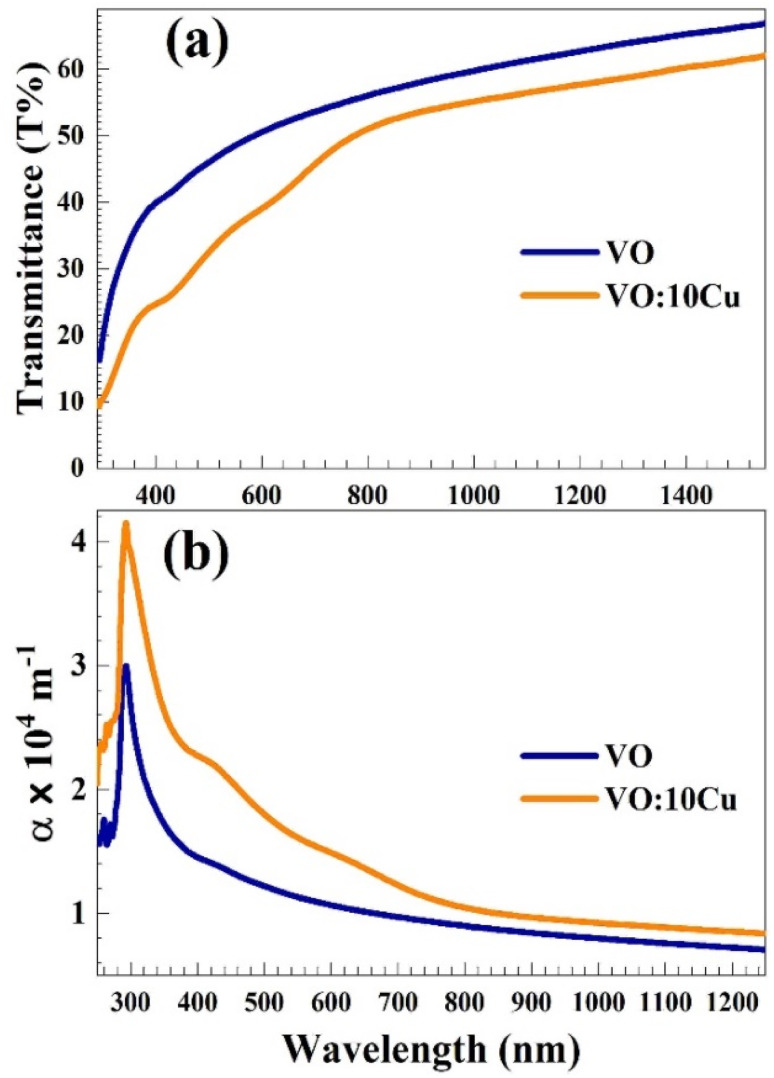
(a) UV-Vis transmittance spectra and (b) absorption coefficient values of the pure and 10 at% Cu-doped V_2_O_5_ films obtained by casting and spin-deposition.

The UV-Vis–NIR spectra (data of transmission in the range of 290–1550 nm) of undoped and 10 at% Cu-doped V_2_O_5_ films, relevant for CO_2_ gas sensing utilization, reveal notable changes in optical behavior upon doping that are shown in Fig. 4. The undoped V_2_O_5_ film exhibits a moderate transmittance ranging from 38% to 56% in the visible region, which increases steadily in the near-infrared wavelengths, reaching up to 67%. In contrast, the Cu-doped V_2_O_5_ film demonstrates a relatively lower optical transparency, with transmittance values of 24–50% in the visible range and extending up to 63% in the infrared region. The absorption coefficient 
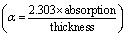
 of the films is shown in Fig. 4b. The absorption peak in the spectra of pure and Cu-doped films at 288 nm and 292 nm, respectively is arising because the absorption of V=O and the associated π → π* electronic transitions,^20^ as discussed in the FTIR results. This band became wider and intense upon Cu-doping. These changes are attributed to Cu incorporation, which modifies the electronic structure and reduces light absorption. A research group suggested that such variations in transmittance are influenced by the dopant's location within the host lattice.^21^

**Fig. 5:**
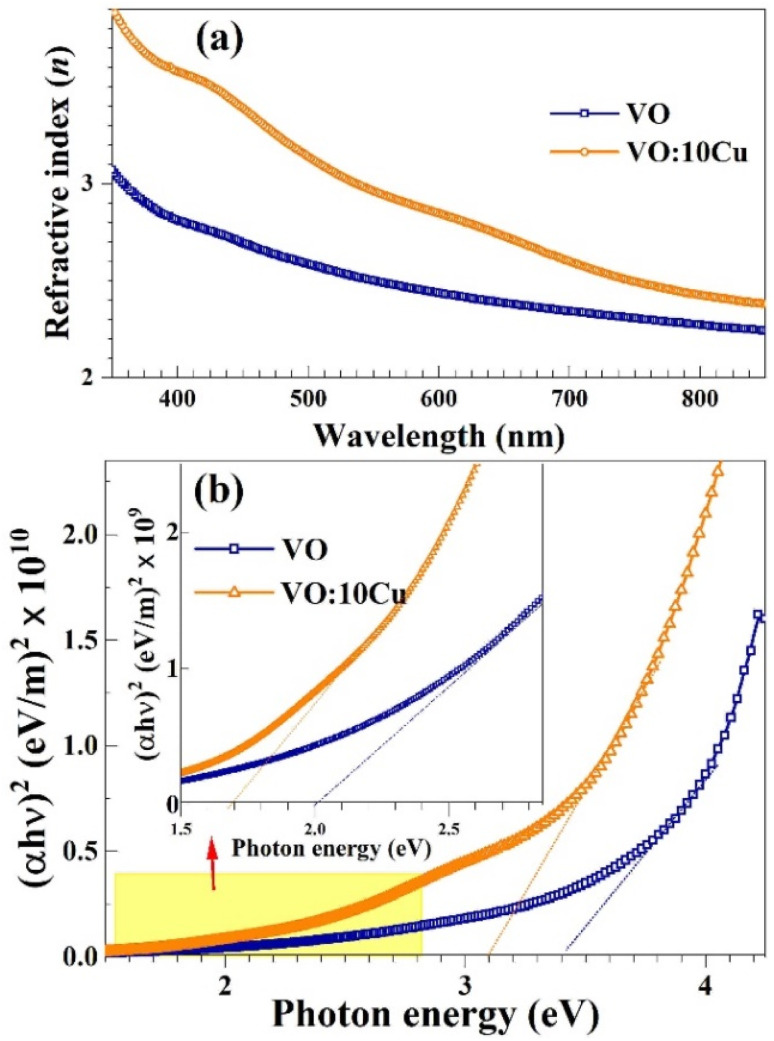
(a) Refractive indices and (b) optical band gaps of the pure and 10 at% Cu-doped V_2_O_5_ films.

The measured reflectance (*R*) was employed to determine the refractive index 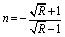
^20^ as shown in Fig. 5a. V_2_O_5_ film has *n* values (in the visible part of the spectra) in the range of 2.88–2.28 with an average value of 2.58 at 500 nm. Cu-doping raises this range to be 3.65–2.45 and the 3.14 at 500 nm. This improvement in the *n* value of the doped film reflects the enhanced film's reflectivity associated with the reduction of *C*_s_ value after Cu incorporation and indicates that the doped film more suitable for optoelectronic applications.^22^

The optical bandgap (*E*_g_) of V_2_O_5_ thin films, which significantly influences their light absorption and interaction with CO_2_ gas molecules, was determined utilizing Tauc's equation:

2 (*αhν*)² = *A*(*hν* − *E*_g_)

where *A* is a constant and *hν* is the photon energy calculated as *hν* (eV) = 1240/*λ* (with *λ* in nm). Fig. 5b presents the (*αhν*)^2^*versus* hν plots for pure and Cu-doped V_2_O_5_ films. The *E*_g_ values were obtained by extrapolating the linear portions of these curves to the photon energy axis (*hν*).^23^ Cu doping was found to reduce the bandgap of V_2_O_5_ from 3.4 eV to 3.1 eV. The inset of the figure indicates possible alternative values of *E*_g_ (2.0 eV and 1.7 eV) for the pure and Cu-doped V_2_O_5_ films. Similarly, Cu-doping reduced the *E*_g_ of the V_2_O_5_ nanosheets prepared by the hydrothermal method from 2.14 and 1.87 eV,^24^ 4 at% Sn decreased the *E*_g_ of the V_2_O_5_ nanoparticles from 2.1 eV to 1.65 eV,^25^ and 1.0 at% Fe reduced the *E*_g_ of the V_2_O_5_ spin-coated film from 2.705 eV to 2.661 eV.^26^ The two optical bandgap values presented in Fig. 5b correspond to different electronic transitions in the V_2_O_5_-based system. The higher-energy gap (3.4 → 3.1 eV) represents the direct allowed transition, which dominates the optical absorption edge and is therefore considered the fundamental optical bandgap of the thin films. The lower-energy feature (2.0 → 1.7 eV) is attributed to indirect transitions and sub-band tail states that arise from oxygen vacancies and localized defect levels introduced by Cu doping.

The reduction in both values upon Cu incorporation reflects the narrowing of the band structure and increased defect density, which enhances visible-light absorption which facilitates improved surface reactivity and charge transfer, that critical parameters for effective CO_2_ gas sensing.

### 3.3 Gas sensing measurements

#### 3.3.1 *I*–*V* characteristic curve and dynamic response

**Fig. 6:**
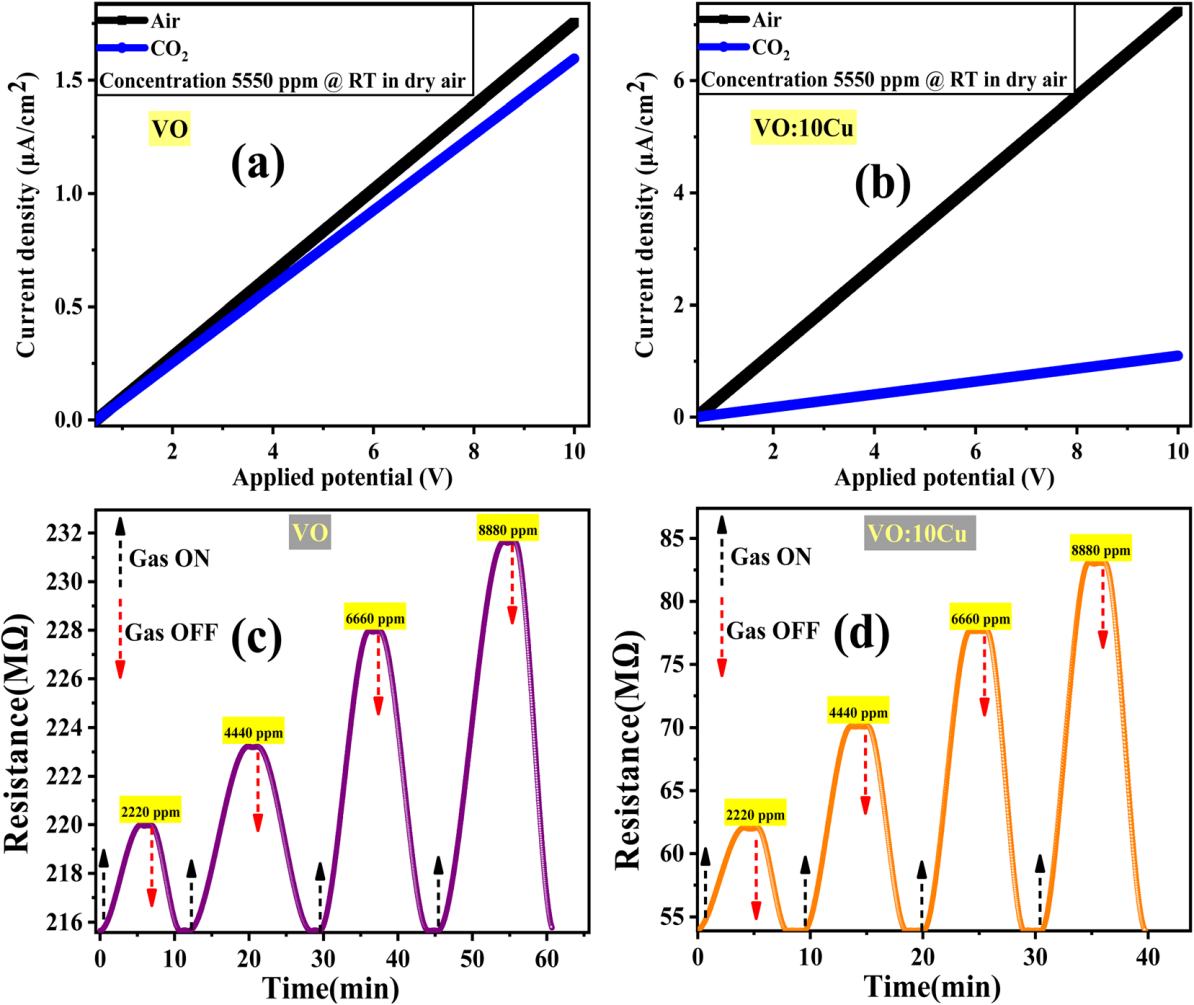
(a) and (b) *I*–*V* characteristic curves, and (c) and (d) dynamic response for pure V_2_O_5_, and 10 at% Cu–V_2_O_5_, respectively.

The *I*–*V* (current–voltage) characteristics of pure V_2_O_5_ and 10 at% Cu-doped V_2_O_5_ were evaluated under ambient conditions in both dry air and in the presence of 5550 ppm CO_2_ at room temperature. As illustrated in Fig. 6a and b, the current measured for both materials decrease noticeably when exposed to CO_2_ compared to air, indicating increased resistance due to gas adsorption. The graphs show that the current is significantly higher in air compared to CO_2_ at the same applied voltages. This behavior indicates that the electrical resistance of pure V_2_O_5_ increases upon exposure to CO_2_. This increase in resistance is a typical response for n-type metal oxide semiconductors like V_2_O_5_, where CO_2_ acts as an oxidizing gas. When CO_2_ molecules are adsorbed on the surface, they interact with adsorbed oxygen species and trap free electrons from the conduction band. This leads to a wider depletion layer and lower conductivity.^27^

The *I*–*V* curve for CO_2_ exposure remains linear, suggesting ohmic contact behavior, but with a lower slope, which confirms a higher resistance in CO_2_. Pure V_2_O_5_ shows a smaller drop in current compared to its Cu-doped counterpart, suggesting higher baseline resistance but lower CO_2_ sensitivity.^28,29^ In contrast, Cu–V_2_O_5_ exhibits a sharp current drop and demonstrates more consistent and linear behavior, which is advantageous for stable sensor response.^22,23^ Cu doping enhances the electronic conductivity of V_2_O_5_ and increases the surface reactivity through the formation of additional oxygen vacancies.^31,32^ These vacancies serve as active sites for gas adsorption and facilitate the redox interactions between CO_2_ molecules and surface-adsorbed oxygen species, which improves sensor performance.^30^

Fig. 6c illustrates the dynamic resistance response of pure V_2_O_5_ when exposed to increasing concentrations of CO_2_ gas (2220 ppm, 4440 ppm, 6660 ppm, and 8880 ppm) at room temperature. The resistance of the material steadily increases with each CO_2_ injection, showing a typical n-type semiconductor behavior where CO_2_ acts as an electron-withdrawing gas. This interaction results in the trapping of conduction band electrons at the surface, which enlarges the depletion layer and increases the overall resistance. The resistance response is reversible with distinct peaks during each exposure, indicating stable adsorption–desorption behavior. However, the magnitude of resistance change is relatively modest, suggesting that pure V_2_O_5_ has limited sensitivity to CO_2_ under these conditions.^25,26^

In contrast, Fig. 6d presents the dynamic resistance response of 10 at% Cu-doped V_2_O_5_ under the same stepwise CO_2_ concentrations. The 10 at% Cu–V_2_O_5_ sample exhibits a significantly greater increase in resistance at each concentration level, reflecting enhanced sensitivity to CO_2_ gas. The resistance peaks are sharper and the recovery between cycles is faster compared to the undoped counterpart, indicating that the doping process has improved the kinetics of gas adsorption and desorption. This enhancement is attributed to the introduction of Cu^2+^ ions into the V_2_O_5_ lattice, which not only modifies the electronic structure but also creates additional oxygen vacancies and active sites. These structural changes promote stronger interaction with CO_2_ molecules and facilitate more efficient charge transfer, resulting in a larger modulation of resistance. Previous studies have shown that Cu doping can improve the electrical conductivity and sensing performance of metal oxides by increasing charge carrier mobility and promoting catalytic activity at the surface.^14,33^ Therefore, 10 at% Cu-doped V_2_O_5_ shows promise as a more effective CO_2_ sensing material compared to its undoped form, particularly for room-temperature applications.

#### 3.3.2 Sensor response and response and recovery times

**Fig. 7:**
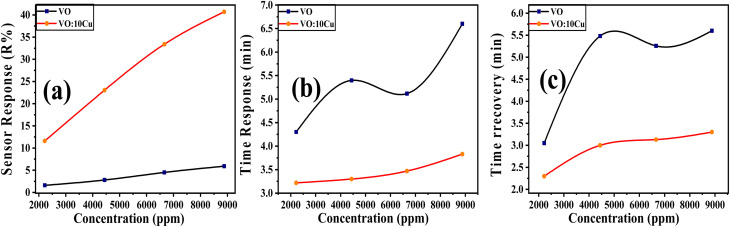
(a) Sensor response, (b) response time, (c) recovery time *vs.* gas concentration for pure V_2_O_5_ and 10 at% Cu–V_2_O_5_, respectively.

Fig. 7a presents the gas sensing response of pure V_2_O_5_ and 10 at% Cu–V_2_O_5_ thin films to varying CO_2_ concentrations ranging from 2220 ppm to 8880 ppm. The sensor response was evaluated using following equation:^34^3
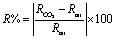
where *R*_CO_2__ and *R*_air_ denote the resistance in CO_2_ and air, respectively. The response of both materials exhibits a positive correlation with gas concentration, indicating an increase in adsorption-driven resistance change. 10 at% Cu-doped V_2_O_5_ shows a markedly higher response compared to pure V_2_O_5_, with the values rising from 11.6% at 2220 ppm to 40.7% at 8880 ppm. In contrast, pure V_2_O_5_ displays a lower response increase, from 1.6% to 5.9% over the same concentration range. This improvement can be attributed to the introduction of Cu dopants, which enhance the electronic conductivity and increase the density of oxygen vacancies and chemisorbed oxygen species on the surface, thereby promoting stronger interactions with CO_2_ molecules.^35,36^

Fig. 7b depicts the response time (*t*_res_) of the sensors, defined as the time required to reach 90% of the maximum resistance change after CO_2_ exposure to different gas concentrations. Both materials exhibit a reduction in response time with increasing CO_2_ concentration. This trend is typical of surface-controlled gas sensors, where higher concentrations accelerate the adsorption kinetics. For pure V_2_O_5_, the response time increases from 4.3 min at 2220 ppm to 6.6 min at 8880 ppm. 10 at% Cu–V_2_O_5_, on the other hand, demonstrates a faster response, increasing from 3.22 min to 3.83 min over the same concentration range. The superior response speed of 10 at% Cu-doped V_2_O_5_ is ascribed to the catalytic effect of Cu, which lowers the activation energy for surface reactions and facilitates quicker charge transfer between adsorbed gas species and the semiconductor matrix.^37,38^

The recovery time (*t*_recov._), defined as the time required for the sensor to return to 90% of its baseline resistance after CO_2_ removal, is shown in Fig. 7c. Both sensors demonstrate a decrease in recovery time with increasing CO_2_ concentration, suggesting faster desorption dynamics at higher surface coverage. Pure V_2_O_5_ exhibits a recovery time increasing from 3.05 min at 2220 ppm to 5.6 min at 8880 ppm, while 10 at% Cu–V_2_O_5_ recovers more rapidly from 2.3 min to 3.3 min across the same range. This enhancement in 10 at% Cu–V_2_O_5_ can be attributed to improved surface reactivity and weaker binding of CO_2_ molecules, facilitating faster desorption. Additionally, Cu doping likely alters the electronic band structure, enhancing the reoxidation process required for recovery.^39–41^ It is noted that the response time is slightly longer than the recovery time for both pure and Cu-doped V_2_O_5_ films, which agrees with earlier reports on metal-oxide CO_2_ sensors, where adsorption-controlled kinetics dominate over the faster desorption step due to the formation of surface carbonate species.

#### 3.3.3 Repeatability, long-term stability, CO_2_ selectivity, and relative humidity

**Fig. 8:**
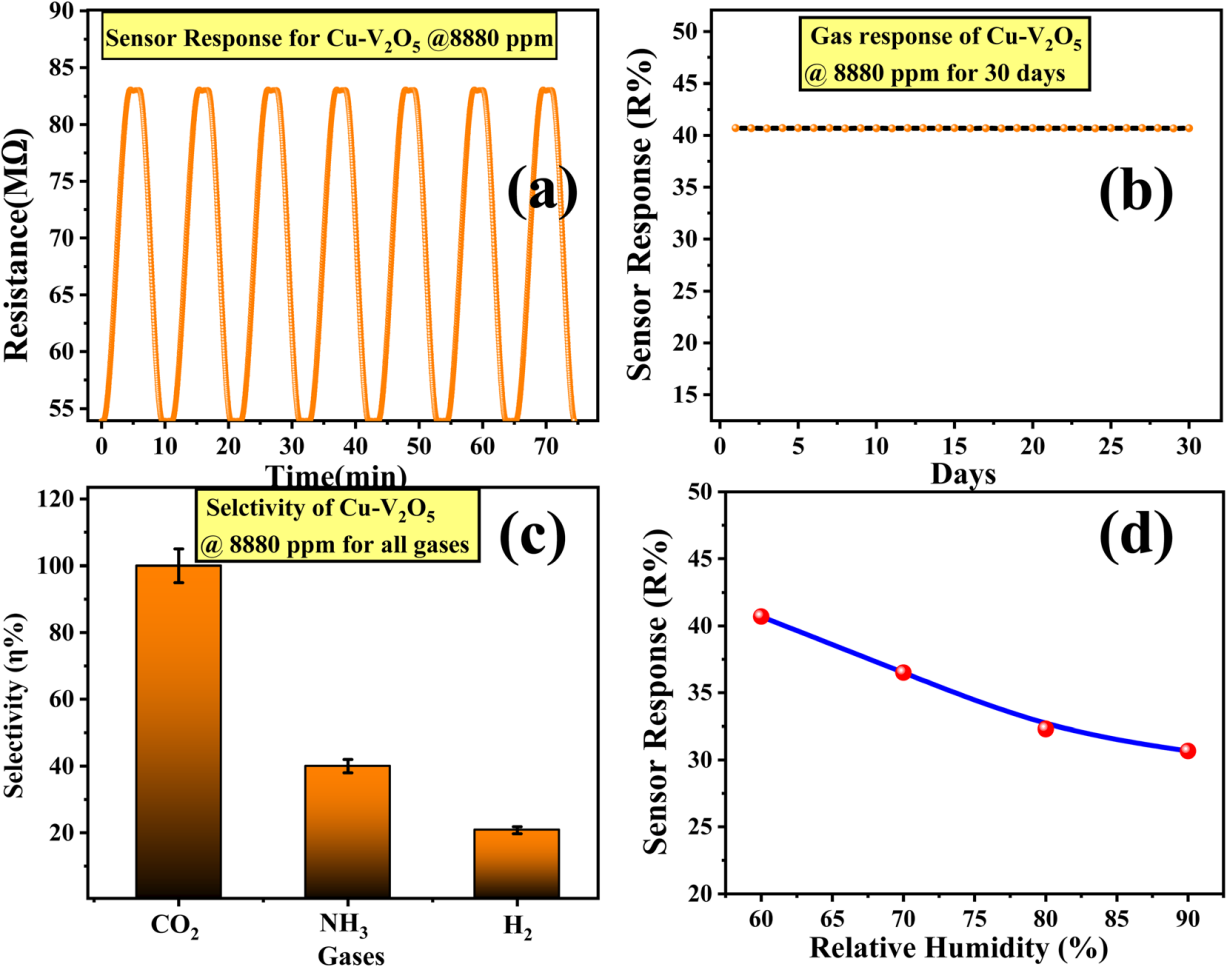
(a) Repeatability, (b) stability, (c) selectivity for several gases at 8880 ppm concentration, RT, and 60% RH, and (d) sensor response *vs.* relative humidity for 10 at% Cu-doped V_2_O_5_ sensor.

The 10 at% Cu–V_2_O_5_ sensor exhibited high repeatability in its response to 8880 ppm CO_2_ over seven consecutive cycles at room temperature and 60% relative humidity, as depicted in Fig. 8a. Long-term stability assessments, illustrated in Fig. 8b, demonstrated a sustained sensor response of approximately 40.7% after 30 days of daily exposure to the same CO_2_ concentration under identical conditions, indicating its operational reliability over extended periods. The sensor's selectivity, quantified by the ratio of its response to CO_2_ compared to interfering gases (H_2_ and NH_3_) according to equation 
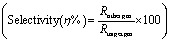
^42^ and shown in Fig. 8c, revealed a significantly higher response towards CO_2,_ with the percentages for H_2_ and NH_3_ being 20.78% and 40%, respectively. It demonstrates that the 10 at% Cu–V_2_O_5_ sensor surface adsorbs more CO_2_ molecules than other gases.

In addition to the CO_2_ sensing results, the influence of humidity on sensor performance was examined, as water molecules are known to interact significantly with the surface of metal-oxide sensors. To evaluate this effect, we monitored the response of the 10 at% Cu-doped V_2_O_5_ film while increasing the relative humidity from 60% to 90% RH under 8880 ppm CO_2_ concentration at RT, shown in Fig. 8d. As the RH increased, a slight reduction in sensor response was observed due to the competitive adsorption between H_2_O and CO_2_ molecules for the same oxygen-vacancy sites. At higher humidity, water molecules tend to form surface hydroxyl groups, which modify surface charge distribution and partially hinder the adsorption of CO_2_, leading to a modest decrease in resistance change. However, despite this suppression, the Cu-doped film maintained a stable and measurable response even at 90% RH, indicating that the higher density of oxygen vacancies created by Cu incorporation helps preserve CO_2_ adsorption capability under moist conditions (Table 1).^43^

**Table 1:** A comparison of gas sensors' CO_2_ detecting capabilities

Nanomaterials	Operating temperature (°C)	Concentration	Sensor response (*R*%)	Response time	Recovery time	References
SnO_2_	240	2000 ppm	1.24	150 s	100 s	44
Co_3_O_4_	150	10 000 ppm	30	227 s	245 s	45
LaOCl–SnO_2_ nanofibers	300	1000 ppm	3.7	130 s	50 s	46
ZnO/CNTs	RT	16 650 ppm	22.4	82.5 s	23 s	47
Ba–CuO	RT	11 100 ppm	9.4	5.6 s	5.44 s	48
SnO_2_–LaOCl nanowires	400	2000 ppm	5.6	57 s	53 s	49
3% Pt–La_2_O_3_/SnO_2_	225	1000 ppm	4.38	—	—	50
ZnO: 4.0 at% La	RT	22 200 ppm	114.22	24.4 s	44 s	51
SnO_2_@CdO	RT	1400 ppm	2.18	45 s	50 s	52
10 at% Cu-doped V_2_O_5_	RT	8880	40.7	3.83 min	3.3 min	This work

##### 3.3.3.1 Gas sensing mechanism

V_2_O_5_ is an n-type semiconductor, characterized by an excess of electrons in its conduction band. The incorporation of Cu dopants into V_2_O_5_ introduces localized energy levels within the bandgap, which enhances the electronic properties of the material.^53^ At room temperature, oxygen (O_2_) molecules adsorb onto the 10 at% Cu–V_2_O_5_ surface, forming negatively charged O species (O_2_^−^, O^−^) by trapping free electrons from the V_2_O_5_ conduction band, through the following reaction:^54^

4 O_2(gas)_ + e^−^ → O_2(ads)_^−^

When CO_2_ gas interacts with the sensor surface, it reacts with the adsorbed O species, releasing the trapped electrons back into the V_2_O_5_'s conduction band. The CO_2_ reaction can also lead to the formation of surface-bound carbonates (CO_3(ads)_^2−^) these carbonates act as insulating layers, impeding electron flow and thereby reducing the conductivity of the material.^55^

5 CO_2(gas)_ + O_2(ads)_^−^ → CO_3(ads)_^2−^ + e^−^

## 4 Conclusion

In this work, pure and 10 at% Cu-doped V_2_O_5_ nanostructure and films were successfully synthesized by a sol–gel/spin-coating method and comprehensively investigated for room-temperature CO_2_ sensing applications. Structural data indicated the formation of pure α-V_2_O_5_ with orthorhombic phase and a strong (001) orientation, while Cu incorporation slightly reduced crystallite size and induced lattice strain without secondary phase formation. FE-SEM and EDX studies revealed dense nanostructured morphologies with uniform Cu distribution and mild oxygen deficiency, both of which are favorable for enhanced surface reactivity. FTIR and UV-Vis analyses further supported these findings, showing characteristic V–O vibrations, *E*_g_ narrowing from 3.4 to 3.1 eV, and increased refractive index from 2.58 to 3.05 at 500 nm, all consistent with improved electronic conductivity and defect-mediated adsorption. Gas sensing measurements demonstrated that Cu doping significantly enhanced sensor performance at room temperature. At 8880 ppm CO_2_, the Cu-doped film achieved a high response of 40.7%, with rapid response (3.83 min) and recovery (3.3 min) times, excellent repeatability, stable operation over 30 days, and selectivity against interfering gases such as H_2_ and NH_3_. These improvements are attributed to the increased oxygen vacancy concentration and modified electronic structure introduced by Cu doping, which enhances charge transfer and gas adsorption kinetics. Overall, this study establishes 10 at% Cu-doped V_2_O_5_ thin films as a promising low-cost and energy-efficient material for ambient CO_2_ detection. The combination of structural stability, optical tunability, and superior sensing performance positions these films as strong candidates for integration into practical environmental and industrial gas monitoring devices. Future work may explore the optimization of doping levels, the effects of humidity, and integration with flexible or microelectronic platforms to advance their applicability in real-world sensing systems further.

## Author contributions

Khaled Abdelkarem: investigation, formal analysis, data curation, conceptualization, writing – original draft, writing – review & editing, Rana Saad: software, methodology, data curation, writing – review & editing, writing – original draft, Adel M. El Sayed: investigation, funding acquisition, formal analysis, data curation, supervision, writing – review & editing.

## Conflicts of interest

The authors declare that they have no conflicts of interest.

## Supplementary Material

RA-016-D5RA07026K-s001

## Data Availability

The data presented in this study are available on request from the corresponding author. Supplementary information (SI) is available. See DOI: https://doi.org/10.1039/d5ra07026k.
