# Supplementary material for: Synthesis of Cu-doped V2O5 thin films with improved optical and CO2 gas sensing
Source: RSC Adv. 2026 Jan 2;16(1):196–207. doi: 10.1039/d5ra07026k (PMC12757838; doi:10.1039/d5ra07026k)
Supplement: RA-016-D5RA07026K-s001 [file RA-016-D5RA07026K-s001.pdf]

Supplementary Information (SI) for RSC Advances.
This journal is © The Royal Society of Chemistry 2025

Supplementary Data

Synthesis of Cu-doped V_2O_5 Thin Films with Improved Optical and CO_2 Gas Sensing

Khaled Abdelkarem[a], Rana Saad[*b], Mohamed Shaban[c], Adel M. El Sayed[d]**

[a] Department of Physics, Chonnam National University, Gwangju 61186, Republic of Korea, oldfighter.khaled123@gmail.com

[b] Department of Physics, Faculty of Science, Beni-Suef University, Beni Suef 62511, Egypt, ranasaad811@gmail.com.

[c] Department of Physics, Faculty of Science, Islamic University of Madinah, P. O. Box: 170, Madinah 42351, Saudi Arabia; mssfadel@aucegypt.edu

[d] Department of Physics, Faculty of Science, Fayoum University, El-Fayoum 63514, Egypt, ams06@fayoum.edu.eg.

Corresponding author[*, **]: ranasaad811@gmail.com; ams06@fayoum.edu.eg

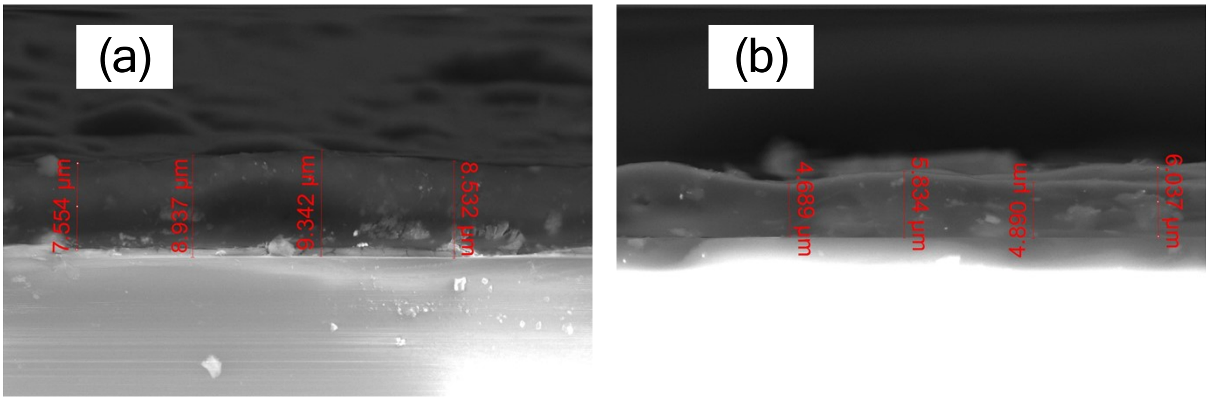

Figure S1: Cross-section of (a) pure V_2O_5 and (b) 10 at.% Cu-doped V_2O_5

The average thickness of the *pure V_2O_5* film is therefore:

$$t_{avg} = \frac{7.554 + 8.937 + 9.342 + 8.532}{4} \approx 8.59\ \mu m$$

Pure V_2O_5 film thickness ≈ 8.6 ± 0.8 µm

The average thickness is:

$$t_{\text{avg}} = \frac{4.689 + 5.834 + 4.890 + 6.037}{4}$$

$$t_{\text{avg}} = \frac{21.45}{4} = 5.36\ \mu m$$

Cu-doped V_2O_5 film thickness ≈ 5.36 ± 0.55 µm

The variation is expected due to spin-coating + sol–gel drying and shrinkage.
